# Identification of Immune-Related Genes *MSR1* and *TLR7* in Relation to Macrophage and Type-2 T-Helper Cells in Osteosarcoma Tumor Micro-Environments as Anti-metastasis Signatures

**DOI:** 10.3389/fmolb.2020.576298

**Published:** 2020-12-14

**Authors:** Zhiyu Chen, Huanhuan Huang, Yang Wang, Fangbiao Zhan, Zhengxue Quan

**Affiliations:** ^1^Department of Orthopedics, The First Affiliated Hospital of Chongqing Medical University, Chongqing, China; ^2^The First Clinical College, Chongqing Medical University, Chongqing, China

**Keywords:** osteosarcoma, metastasis, tumor immune infiltration cell, macrophages, type-2 T-helper cell, MSR1, TLR7

## Abstract

Metastasis of osteosarcoma (OS) is an essential factor affecting the prognosis and survival of patients. The tumor microenvironment, including tumor immune-infiltrating cells (TIIC), is closely related to tumor progression. The purpose of this study was to investigate the differences between metastatic and non-metastatic immune-infiltrating cells in OS and to identify key immune-related genes. The differences in immune infiltration in OS metastasis were calculated based on the ssGSEA algorithm of 28 immuno-infiltrating cells. Weighted gene co-expression network analysis (WGCNA) and intersection analysis were used to screen immune-related modules and hubgenes. Univariate/multivariate/Lasso Cox regressions were used for models construction and signatures screening. The receiver operating characteristic (ROC) and Kaplan–Meier (K–M) curves were constructed to observe the metastases of different groups. Both internal and external data were verified. We found that macrophages and Type-2 T-helper cells were significantly decreased in patients with OS metastases. The high-risk groups obtained from multivariate/Lasso Cox models constructed with 11 immune-related hubgenes almost all underwent distant metastases within 5 years. Interestingly and importantly, two genes, *MSR1* and *TLR7*, appeared in various models and various hubgenes, which play an anti-metastasis role and may prolong overall survival in OS. Our study may help elucidate the impact of TIIC on OS metastasis outcomes and to identify biomarkers and therapeutic targets.

## Introduction

As a primary malignant bone cancer, osteosarcoma (OS) most commonly affects children and adolescents. The application of multi-drug chemotherapy regimens after 1970 increased long-term survival rates to about 70 percent in local OS patients. However, the prognosis of OS is still not optimistic because of the less than 25% 5-year survival rate for recurrent or metastasis (Mirabello et al., 2009; Kansara et al., 2014; Isakoff et al., 2015). Distant metastasis of OS has puzzled patients and clinicians for decades. Therefore, it is of great importance to explore the mechanism underlying OS metastasis and develop effective therapies to reduce metastasis and improve survival.

The tumor microenvironment (TME) plays important role in tumor development, metabolism, and metastasis. The TME, comprising stromal cells, fibroblasts, and immune cells, is complex and continuously evolving since tumors release several factors to reprogram the surrounding cells to establish the microenvironment (Hinshaw and Shevde, 2019). The distributions of different types of tumor-infiltrating immune cells (TIIC) have different impacts on the progression of tumors according to cancer type and patient status, such as metastasis (Pearce et al., 2018). In OS, the crosstalk between TIIC and cancer cells leads to an immunosuppressive environment: infiltrating T-lymphocytes are reduced and immune responses are limited (Heymann et al., 2019). Innate immune cells such as macrophages communicate with metastatic OS cells within the TME via exosomes, which produces a more tumor-permissive environment to protect the tumor from immune-mediated killing (Wolf-Dennen et al., 2020). The emergence of high-throughput sequencing technology and the development of bioinformatics have made the contributions of TIIC clearer.

At present, a few studies have reported osteosarcoma-associated immune infiltration. Zhang et al. (2020) used ESTIMATE to investigate the immune score of 22 TIIC in OS. Zhang et al. (2019) preliminarily identified metastatic signatures for lung metastasis and survival. To the best of our knowledge, however, few studies have investigated TIIC associated with human OS metastasis. In this study, OS expression data and clinical information were downloaded from Therapeutically Applicable Research to Generate Effective Treatments–Osteosarcoma (TARGET-OS) and Gene Expression Omnibus (GEO) databases. Single sample Gene Set Enrichment Analysis (ssGSEA) (Barbie et al., 2009), using lists of pan-cancer immune metagenes generated by Charoentong et al. (2017), was applied to calculate the immune infiltration of 28 TIIC subsets in metastatic OS.

Weighted gene co-expression network analysis (WGCNA) and univariate/multivariate/Lasso Cox regression constructions were used to identify metastatic immune-related signatures. Importantly, two genes, *MSR1* and *TLR7*, which are related to metastasis (Met), macrophages (MA), and type-2 T-helper cell (Th2), appeared in various models and hubgenes. The results of our study will help to evaluate the metastatic possibility in OS patients, enable doctors and patients to make early response measures, and provide new potential targets and strategies for new therapies for the metastatic OS.

## Materials and Methods

### Data Acquisition and Filtration

The level-3 RNA-seq data of 88 osteosarcoma patients and 87 matching clinical matrix about metastasis information (containing 32 metastatic samples and 55 non-metastatic samples) in TARGET-OS datasets were downloaded using Xena Platform (Goldman et al., 2020)^1^. Two OS GEO microarray datasets were downloaded using R language (version 3.6.3) via R package GEOquery (version 2.54.1). GSE21257 included 53 pre-chemotherapy OS biopsy samples with 34 metastatic and 19 non-metastatic samples with metastatic status and metastatic time information. GSE42352 consisted of 19 high-grade OS cell lines, 84 high-grade OS pre-chemotherapy biopsies (containing 53 samples with OS metastasis information), 12 mesenchymal stem cell samples, and three osteoblast cell samples. Only RNA-seq or microarray samples that provided matching metastasis information were chosen in our study.

### Single Sample Gene Set Enrichment Analysis (ssGSEA) and Selection of TIIC Subsets

Immune cell marker gene expression information of 28 subgroups was obtained from the article published by Charoentong et al. (2017). In order to determine the distribution of TIIC subsets which contributes OS metastasis, TARGET-OS, and GSE21257 datasets were used to construct ssGSEA (Subramanian et al., 2005) using the R package GSVA (version 1.34.0) based on these immune cell marker genes. Then normalized enrichment score (NES) of each TIIC subsets were calculated and visualized via R packages pheatmap (version 1.0.12), ggplot2 (version 3.3.0), and ggpubr (version 0.3.0). *Q*-value < 0.05 was set to multiple-test adjust for *P*-values based on R package fdrtool (version 1.2.15). The NESs of TIIC subsets with significant differences (*P*-value < 0.05, *Q*-value < 0.05, Wilcoxon test) were used for the following analysis.

### Weighted Gene Co-expression Network Analysis (WGCNA)

R package WGCNA (Langfelder and Horvath, 2008) (version 1.69) was applied to screen metastasis-related, selected TIIC-subset-related modules and hubgenes (Zhang and Horvath, 2005). After detecting and excluding outliers via hierarchical cluster analysis, samples were clustered by the average method to observe metastatic and selected TIIC subsets’ traits. 87 TARGET-OS samples and 52 GSE21257 samples were used to construct scale-free topology networks, respectively. All gene adjacencies were calculated to make a topological overlap matrix (TOM) and corresponding dissimilarity (1-TOM). Modules with correlation coefficients greater than 0.8 were merged. The clinical features of samples and module-trail relationships were demonstrated based on metastasis-trait and NESs of selected TIIC-subsets. The highest correlated modules with statistical significance (*P* < 0.05) were, respectively, selected in TARGET-OS and GSE21257. Module membership, and gene significance were constructed for different trait-related hubgenes selection with module membership >0.8, gene significance >0.2, and *Q*-value < 0.01. Point-biserial correlation was used for dichotomous variables, and spearman correlation was used for continuous variables. The statistical method was used student *T*-test.

### Gene Enrichment Analyses and Hubgenes Intersected Analysis

Selected module genes in GSE21257 and TARGET-OS were intersected via R package VennDiagram (version 1.6.20). Gene Ontology (GO) and Kyoto Encyclopedia of Genes and Genomes (KEGG) enrichments were conducted based on intersected module genes by R package clusterProfiler (Yu et al., 2012) (version 3.14.3). The intersection of different trait-related hubgenes in TARGET-OS and GSE21257 and immune cell marker genes were constructed via R package UpSetR (Conway et al., 2017) (version 1.4.0). Heatmaps were used to display intersected immune-related metastatic hubgenes expression in different samples via R package pheatmap.

### Construction and Validation of Metastasis Signature

Univariate/multivariate Cox regression and Lasso Cox regression analyses were performed to screen out which immune-related genes play an important role in prognoses of OS metastasis. Because only GSE21257 contains metastasis status and time information, it was considered to build metastasis models based on the multivariate Cox regression and Lasso Cox regression using R package glmnet (version 4.0) and survival (version 3.1-12). Kaplan–Meier (K–M) curve and forest plot were constructed by package survminer (version 0.4.6). R package survivalROC (version 1.0.3) was used to build time-dependent receiver operating characteristic (ROC) curves. Metastasis status, metastasis time, risk score, and relevant gene expressions were visualized by R package pheatmap, ggplot2, and ggpubr (version 0.3.0). *Q*-value < 0.05 was used for multiple inspection corrections of gene expression via R package fdrtool.

## Results

**FIGURE 1:**
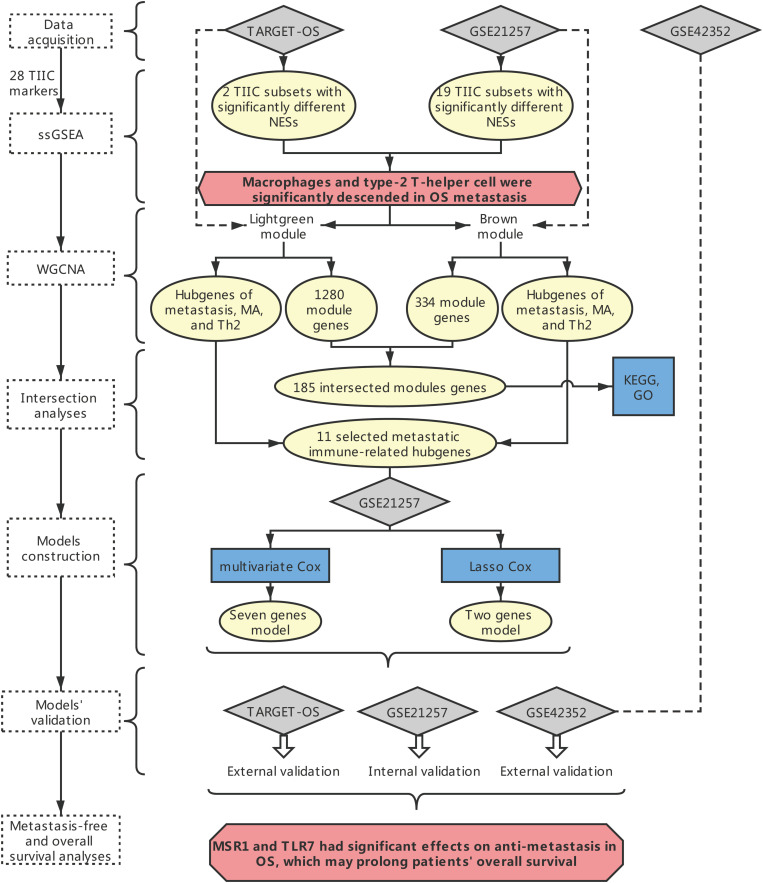
The overall framework. The dotted box represents the main method. The blue boxes represent some specific analysis methods. The gray diamond represents the dataset. The pale-yellow circles represent the results of the analyses. The red hexagon represents the main conclusion.

The overall framework was shown in Figure 1.

### Distribution of TIIC NESs With Metastasis versus Non-metastasis in OS

**FIGURE 2:**
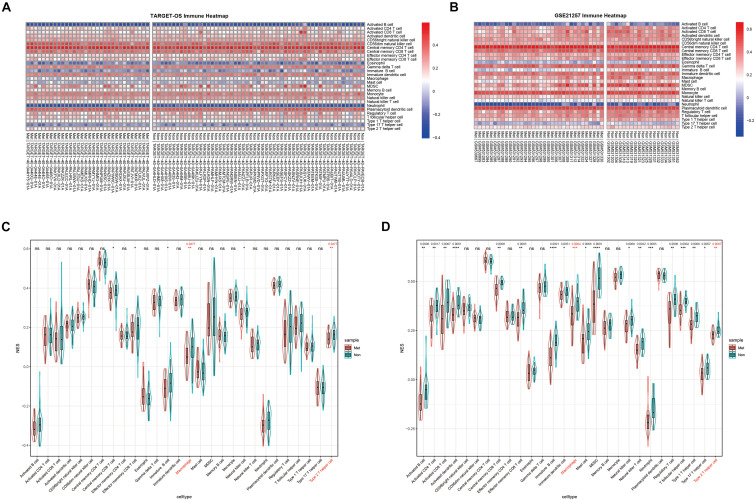
The distribution of 28 TIIC subsets with metastasis or non-metastasis in OS. **(A,B)** Immune heatmap presents NESs of 28 TIIC subsets in each OS sample in TARGET-OS **(A)** and GSE21257 **(B)**. **(C,D)** The NES differences of 28 TIIC subsets between metastasis and non-metastasis in TARGET-OS **(C)** and GSE21257 **(D)**. Significant *Q*-values (<0.05) were shown above the asterisk of significant *P*-values. Significance levels: ****P < 0.0001; ***P < 0.001; **P < 0.01; *P < 0.05; ns, no significance.

Normalized enrichment score is also called the normalized enrichment statistic (Hänzelmann et al., 2013). The higher one sample’s NES of a TIIC subset, the more immune infiltration of associated TIIC type. Immune-infiltrating NESs were calculated through ssGSEA using immune marker genes made by Charoentong et al. (2017). As shown in Figures 2A,B, immune heatmaps revealed every TIIC-related NES distribution in every OS patient with metastasis or non-metastasis. Based on TARGET-OS (Figure 2C) and GSE21257 (Figure 2D), metastasis-related immune NESs’ differences of OS metastasis (Met) and non-metastasis (Non) were displayed among 28 immune subgroups. There were only two TIIC subsets, macrophage and type 2 T-helper cell, with *P*-value and *Q*-value statistical significantly different NESs in both TARGET-OS (RNA sequencing data) and GSE21257 (RNA microarray data), which were both decreased in metastatic OS.

### Selection of the Most Relevant Module and Genes via WGCNA

**FIGURE 3:**
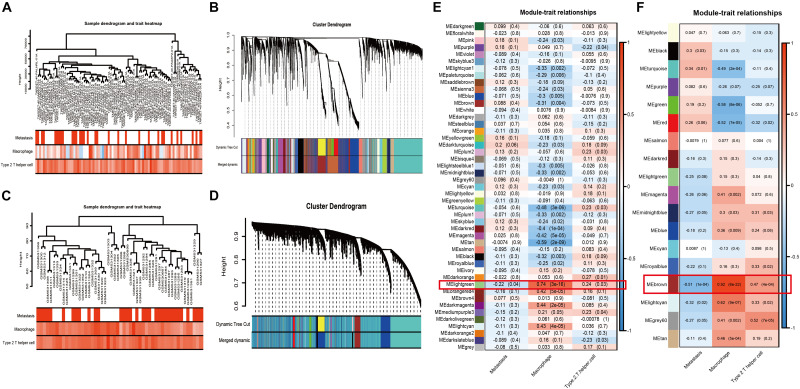
WGCNA and module selection. **(A,C)** Sample dendrogram and trait heatmaps with metastasis, MA, and Th2 of TARGET-OS **(A)** and GSE21257 **(C)**. In metastasis-trait, red represents patients with metastasis and white represents without metastasis; in MA and Th2-traits, the red or blue deeper, the positive or negative NESs higher. **(B,D)** Cluster dendrograms in TARGET-OS **(B)** and GSE21257 **(D)**. **(E,F)** Heatmaps of module–trait relationships between gene modules and traits of metastasis, MA, and Th2 in TARGET-OS **(E)** and GSE21257 **(F)**. The number in brackets on the right of each cell is the *P*-value, and the number on the left is the correlation coefficient.

Weighted gene co-expression network analysis, as a systems biology approach to describe the correlation patterns among genes, was used to determine significantly relevant modules and hubgenes. The sample dendrogram and trait heatmap demonstrated the samples’ metastatic status, MA, and Th2 immune infiltration (Figure 3A: TARGET-OS; Figure 3C: GSE21257). Scale-free topology networks were built to classify all genes into biological modules and to screen out hubgenes. In our study, the soft threshold (β) was set at 3 (scale *R*^2 = 0.93) to establish a scale-free topology based on TARGET-OS (Supplementary Figures 1A–D). Meanwhile, β was set at 2 (scale *R*^2 = 0.91) based on GSE21257 (Supplementary Figures 1F–I). Modules with a correlation coefficient greater than 0.8 were merged. Subsequently, 47 modules in TARGET-OS (Figure 3B) and 18 modules in GSE21257 (Figure 3D) were set up. Next, module-trait relationships’ heatmaps were demonstrated as shown in Figure 3E (TARGET-OS) and Figure 3F (GSE21257). Interestingly, the most negatively correlated module of TARGET-OS in metastasis-trait (correlation coefficient = −0.22), lightgreen, which is the only statistically significantly different module (*P* = 0.04), was the module most positively correlated with MA-trait (correlation coefficient = 0.74, *P* < 0.001). And this module also had a second significantly (*P* = 0.03) positive correlation coefficient (0.24) with Th2-trait. Similarly, brown, the most negatively significantly correlated module with metastasis (correlation coefficient = −0.51, *P* < 0.001), was also the most positive module correlated with MA-trait (correlation coefficient = 0.92, *P* < 0.001) and the second positive correlated with Th2-trait (correlation coefficient = 0.47, *P* < 0.001). Finally, gene significances of three different traits versus module membership are displayed in Supplementary Figures 2A–C (GSE21257) and Supplementary Figures 2D–F (TARGET-OS).

### Enrichment of Intersected Module Genes and Identification of Metastatic Immune-Related Hubgenes

**FIGURE 4:**
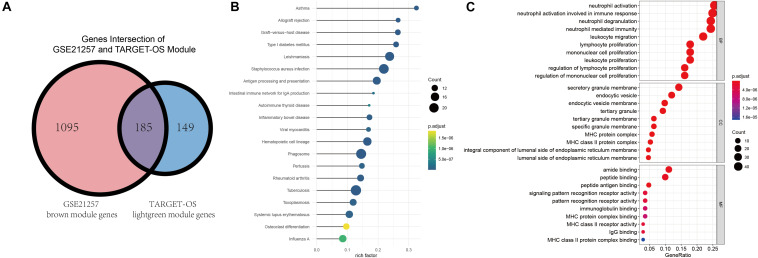
Intersection of selected modules’ genes, KEGG, and GO enrichment analyses. **(A)** The intersection of brown module of GSE21257 and lightgreen module of TARGET-OS. **(B)** Kyoto Encyclopedia of Genes and Genomes (KEGG) enrichment was demonstrated via bubble chart. **(C)** Gene Ontology (GO) was presented through biological process (BP), cellular component (CC), molecular function (MF).

Gene ontology and KEGG were conducted to identify these intersected immune-related genes’ functions and pathways. Before enrichment analyses, Venn plot of genes in brown module of GSE21257 (*n* = 1280) and lightgreen module of TARGET-OS (*n* = 334) is demonstrated that there were 185 genes were intersected (Figure 4A). As displayed in Figure 4B, all results in KEGG were associated with immunologic process. As GO results demonstrated in Figure 4C, neutrophil activation, neutrophil activation, and neutrophil degranulation were the top three enriched terms in biological process (BP). Secretory granule membrane, endocytic vesicle, and endocytic vesicle were the top three enrich terms in cellular component (CC). And the top three terms in molecular function (MF) were amide binding, peptide binding, and peptide antigen binding. In addition to the top three terms in BP, CC, and MF, many immune-related terms were enriched, which indicated that these 185 genes play important roles in various immune processes.

**FIGURE 5:**
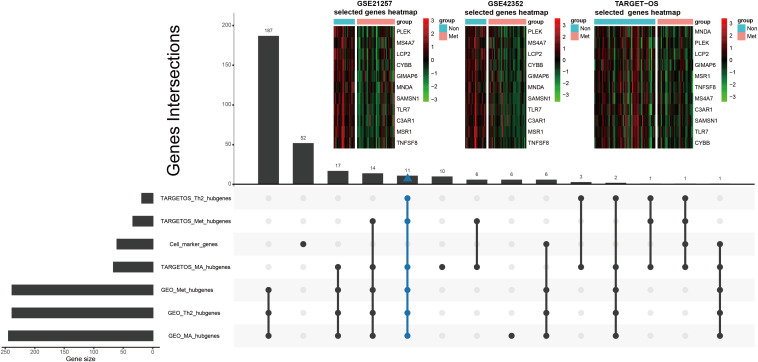
Further selection of metastatic immune-related hubgenes in TARGET-OS and GSE21257. Excluding TIIC marker genes, eleven hubgenes were intersected of traits of metastasis (Met), macrophage (MA), and type 2 T helper cell (Th2) in TARGET-OS and GSE21257, which is shown in the lower part of this Figure. The 11 selected metastatic immune-related hubgenes’ expressions in GSE21257, GSE42352, and TARGET-OS are displayed in the upper part of this Figure.

Tumor immune-infiltrating cells marker genes were considered immune genes. Genes that are associated with TIIC subsets and excluding TIIC cell markers are considered immune-related genes. Hubgenes among metastasis-trait, MA-trait, and Th2-trait in two different datasets were intersected without immune cell marker genes, which screened out 11 immune-related hubgenes as shown in the low part in Figure 5. And displayed in the upper part of Figure 5, heatmaps demonstrate the 11 selected immune-related hubgenes’ expression in every sample of different datasets (GSE21257: microarray data; GSE42352: microarray data; TARGET-OS: RNA-seq data). The differentially expressed profiles of these 11 hubgenes were shown in Supplementary Figures 3A–C and all the 11 hubgenes were included in 185 immune-related genes (Supplementary Figure 3D). Subsequently, the 11 selected hubgenes were used to conducted prognostic models to find out their prognostic implications.

### Univariate and Multivariate Cox Regression Analyses for OS Metastatic Immune-Related Signature Construction

GSE21257 was chosen to construct the OS metastatic immune-related prediction model because only it has expression data with metastatic status and metastatic time information. And it is worth noting that samples in GSE21257 were all pre-chemotherapy biopsies with OS. Ten of the 11 immune-related hubgenes (except *MNDA* with *P* = 0.098) were significantly associated with OS metastasis via univariate Cox regression analysis. However, all the 11 immune-related hubgenes were statistically significant with the log-rank test (Table 1). Seven genes (including *MS4A7, LCP2, MNDA, MSR1, TLR7, SAMSN1, C3AR1*) were identified in the multivariate Cox analysis. The formula is as follows: (0.6685 × *MS4A7* expression value) + (0.8025 × *LCP2* expression value) + (0.7447 × *MNDA* expression value) + (−2.5328 × *SAMSN1* expression value) + (−2.3244 × *TLR7* expression value) + (2.8589 × *C3AR1* expression value) + (−2.0363 × *MSR1* expression value).

**TABLE 1:** Univariate Cox and log-rank test.

Gene ID	HR	95% CI	*P*.value	*P*.value of log-rank
C3AR1	0.31	(0.14–0.7)	0.0046	5.20 × 10^–06^
CYBB	0.51	(0.34–0.76)	0.001	0.000715
GIMAP6	0.36	(0.19–0.67)	0.0012	9.19 × 10^–06^
LCP2	0.57	(0.39–0.84)	0.0049	0.001965
MNDA	0.71	(0.47–1.1)	0.098	0.033169
MS4A7	0.59	(0.41–0.86)	0.0057	0.000235
MSR1	0.16	(0.064–0.4)	8.20 × 10^–05^	6.02 × 10^–07^
PLEK	0.61	(0.43–0.88)	0.0081	0.005778
SAMSN1	0.33	(0.17–0.65)	0.0013	4.73 × 10^–06^
TLR7	0.36	(0.21–0.62)	2.00 × 10^–04^	1.35 × 10^–05^
TNFSF8	0.037	(0.0026–0.53)	0.015	0.001496

**FIGURE 6:**
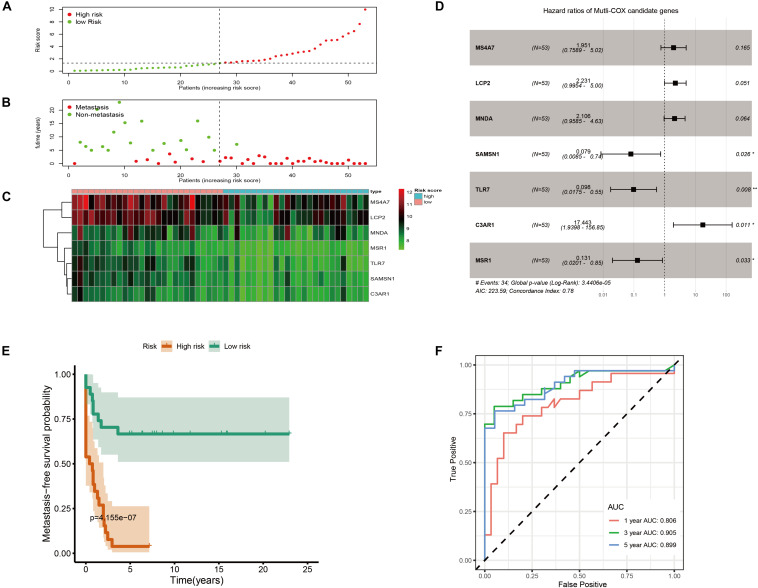
Immune-related metastatic risk model based on GSE21257 constructed by multivariate Cox. **(A)** Dot plot of risk score. High-risk and low-risk samples were represented using red and green colors, respectively. **(B)** Dot plot of metastasis status in OS. The ordinate represents the time when the metastasis occurs. Red and green represent metastasis and non-metastasis cases, respectively. **(C)** Heatmap of high (blue) and low (red) risk score groups of seven multivariate Cox candidate genes. **(D)** Forest plot displays Hazard ratios of seven multivariate Cox candidate genes to metastasis-free survival. **(E)** K–M curve represents that OS patients with high risk had significantly worse metastasis-free survival probability. **(F)** Time-dependent ROC curves of metastasis-related multivariate Cox model.

As shown in Figure 6A, metastatic immune-related prediction model distribution was cut off by the median value of risk score into high- and low-risk groups. Nearly all people in the high-risk group (except one sample) had distant metastases within 5 years (Figure 6B). The heatmap showed the conditions of the expression of the seven genes (Figure 6C). The forest plot is shown in Figure 6D. The high-risk group had a significantly worse metastasis-free survival probability with *P* = 4.155 × 10^–7^ (Figure 6E). Time-dependent ROC curves were demonstrated that the multivariate Cox model had good robustness with 1-year AUC of 0.806, 3-year AUC of 0.905, and 5-year AUC of 0.899 (Figure 6F). Metastasis-free survival of seven genes was shown in Supplementary Figure 4.

### Lasso Cox Regression Analysis for Further Identification of OS Metastatic Immune-Related Signature

**FIGURE 7:**
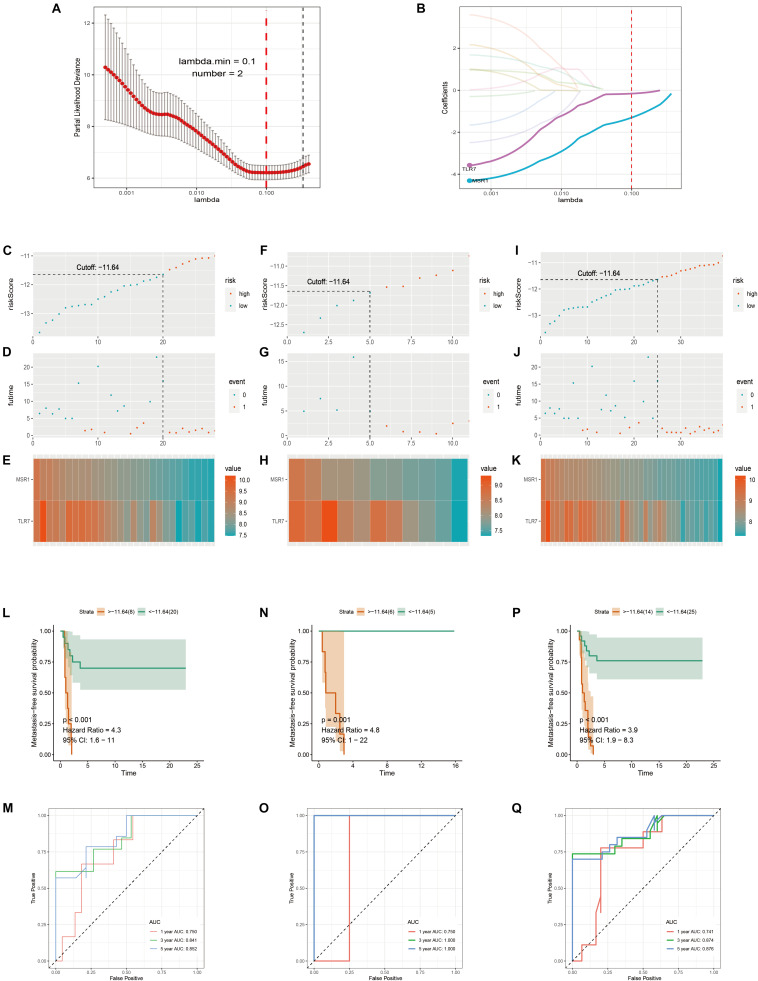
Immune-related metastatic risk model based on GSE21257 constructed by Lasso Cox. 10-fold cross-validation Lasso regression, using minimum likelihood deviance to get 2 best immune-related metastatic genes, MSR1, TLR7 **(A,B)**. Dot plots of risk scores were demonstrated in the training cohort **(C)**, testing cohort **(F)**, and total cohort **(I)**. Red color represents the sample with high risk and blue represents the sample with low risk. Dot plots of metastasis status were shown in **(D)** (training cohort), **(G)** (testing cohort), and **(J)** (total cohort). Red represents the sample with metastasis and blue represents the sample with non-metastasis. **(E,H,K)** The two genes’ expressions in the training, testing, and total cohorts were shown, respectively. Metastasis-free survival K–M curves of Lasso Cox model in train cohort **(L)**, testing cohort **(N)**, and total cohort **(P)**. Red lines represent patients with high-risk scores and green with low-risk scores. Time-dependent ROC curves of train cohort **(M)**, testing cohort **(O)**, and total cohort **(Q)**. The red, green, and blue lines, respectively, represent AUC values in predicting the 1-year, 3-year, and 5-year metastasis-free survival.

Samples in GSE21257 were randomly divided into training and testing cohorts by a ratio of 7–3. Lasso Cox regression analysis was used to build a metastatic immune-related model in the training cohort (Figure 7A). Then a two-gene-based (*MSR1*, *TLR7*) classifier was constructed as shown in Figure 7B. The formula of Lasso Cox model is as follows: (−0.1589 × *TLR7* expression value) + (−1.3040 × *MSR1* expression value). Samples were divided into high- and low-risk groups by using a risk score cutoff of −11.64 (training cohort: Figure 7C; testing cohort: Figure 7F; total cohort: Figure 7I). As displayed in Figure 7D (training cohort), Figure 7G (testing cohort), and Figure 7J (total cohort), all patients with high-risk scores developed OS metastases within 5 years compared to the low-risk group. Figures 7E,H,K demonstrated the expression of the two genes in training, testing, and total cohorts, respectively. The hazard ratio of *TLR7* (0.293, *P* = 0.004) and *MSR1* (0.105, *P* = 0.004) demonstrated these two genes played anti-metastatic roles in OS (Supplementary Figure 5). Metastasis-free survival-related K–M curves in training cohort (Figure 7L), testing cohort (Figure 7N), and total cohort (Figure 7P) indicated that OS patients with high-risk scores had significantly worse metastasis-free survival comparing with low-risk score patients. curves were made to show the robustness of the Lasso Cox model. As displayed in Figures 7M,O,Q, AUC of Time-dependent ROC in the training cohort, testing cohort, and total cohort, respectively, described the Lasso predict model has good robustness in 1-year, 3-year, and 5-year.

### Internal and External Validation of Both Models

**FIGURE 8:**
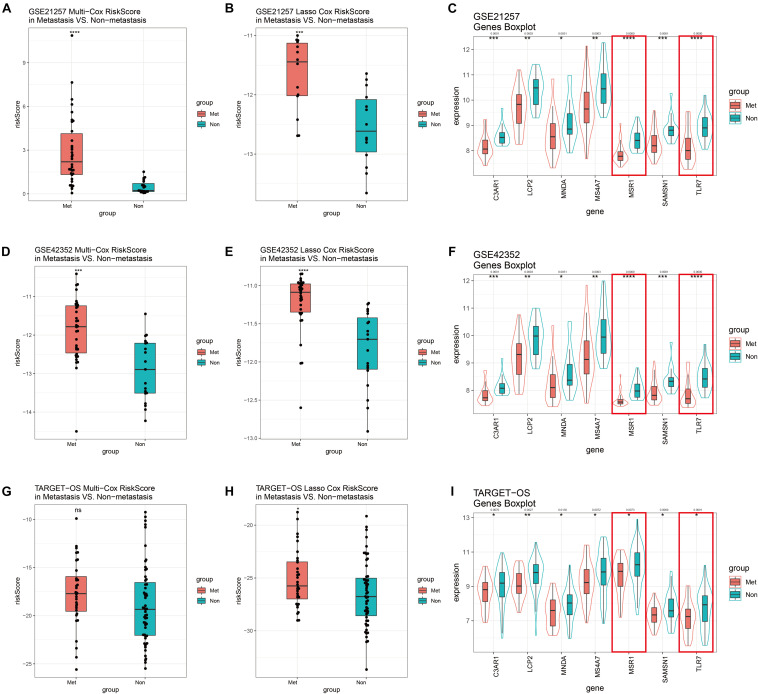
GSE21257 internal and GSE42352/TARGET-OS external validation of multivariate Cox and Lasso Cox models. Risk scores of the multivariate Cox model in GSE21257 **(A)**, GSE42352 **(D)**, and TARGET-OS **(G)**. Patients with metastases had higher risk scores. Risk scores of the Lasso Cox model in GSE21257 **(B)**, GSE42352 **(E)**, and TARGET-OS **(H)** display metastatic OS patients had higher Lasso Cox risk scores. Expression levels with seven multivariate Cox candidate genes in GSE21257 **(C)**, GSE42352 **(F)**, and TARGET-OS **(I)**, in which the red boxes represent expressions of two Lasso-Cox-derived metastatic immune-related genes, *MSR1* and *TLR7 Q*-value less than 0.05 were shown above the asterisk of significant *P*-value. Significance levels: ****P < 0.0001; ***P < 0.001; **P < 0.01;*P < 0.05; ns, no significance.

GSE21257 was used to perform internal validation, while GSE42352 and TARGET-OS were used to perform external validation. Risk scores of multivariate Cox model of metastatic groups were significantly higher than non-metastasis in GSE21257 (Figure 8A) and GSE42352 (Figure 8D). However, there was no statistically significant difference in risk scores of the multivariate Cox model in TARGET-OS (Figure 8G). In the Lasso Cox model, risk scores of the metastatic samples in TARGET-OS were significantly different higher with non-metastatic samples (Figure 8H). Meanwhile, as shown in Figures 8B,E, Lasso Cox risk scores were highly significantly different between metastasis and non-metastasis in GSE21257 and GSE42352. The seven multivariate-Cox-derived genes are displayed in Figures 8C,F,I, and all of them were decreased significantly in metastatic groups, which Lasso-Cox-derived genes, *MSR1* and *TLR7* were included in these Figures.

### Metastasis-Free and Overall Survival Analyses of *MSR1* and *TLR7*

**FIGURE 9:**
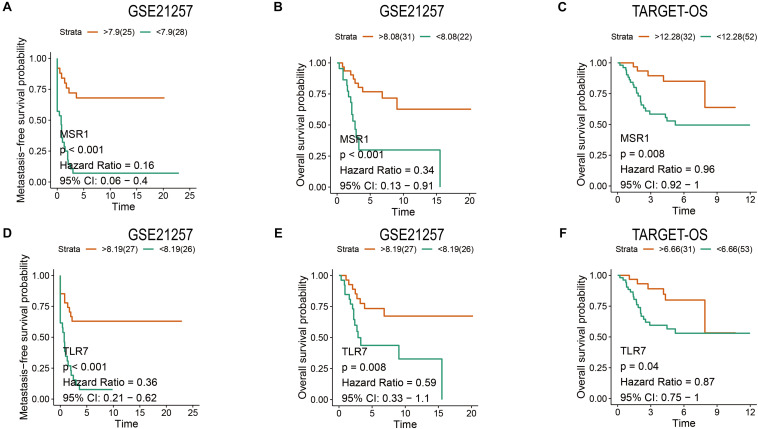
The metastasis-free and overall survival analyses of *MSR1* and *TLR7*. The metastasis-free survival analyses of *MSR1* **(A)** and *TLR7* **(D)** based on GSE21257. The overall survival analyses of *MSR1* **(B)** and *TLR7* **(E)** based on GSE21257, and *MSR1* **(C)** and *TLR7* **(F)** based on TARGET-OS.

Patients have a very poor prognosis in metastatic OS (Meazza and Scanagatta, 2016). Metastatic survival analyses based on GSE21257, overall survival analyses based on GSE21257 and TARGET-OS were conducted to verify the impact of *MSR1* and *TLR7* on OS metastatic and overall survival. As shown in Figures 9A,D, patients with low expression of *MSR1* or *TLR7* had significantly poor metastasis-free survival. As displayed in Figures 9B,C, higher *MSR1* expression indicated patients had better overall survival. And as displayed in Figures 9E,F, OS patients with better overall survival were with higher *TLR7* expression. Therefore, *MSR1* or *TLR7* were conducive to improving metastasis-free survival probability and prolonging overall survival in OS.

## Discussion

Osteosarcoma is a primary malignant bone cancer affecting children, adolescents, and young adults (Mirabello et al., 2009). Metastasis remains a challenge for doctors and patients, which is the major factor affecting the OS patients’ survival (Isakoff et al., 2015). High-throughput omics analysis has increasingly applied to osteosarcoma with the rapid innovating of sequencing technology and bioinformatics (Zhao et al., 2020). Researchers aim to use these techniques to discover the causes of tumorigenesis (Mirabello et al., 2020), metastasis (Xie et al., 2018), drug resistance (Zhu et al., 2019), and other disease processes, and to identify prognostic biomarkers (Zhang et al., 2019; Feng et al., 2020; Tu et al., 2020).

Since James P. Allison and Tasuku Honjo shared the 2018 Nobel Prize in Physiology or Medicine for discovering a cancer cure via the suppression of negative immune regulation (The Nobel Prize, 2020), research and applications of TIIC in tumors have reached a new climax. Recently, Wu et al. (2020) defined three broad immune subgroups with low, medium, and high levels of immune infiltration with osteosarcoma. Zhang et al. (2020) investigated 22 TIIC and immune genes in OS. Scott et al. (2018) developed a novel Gene Cluster Expression Summary Score (GCESS), and GCESS quantified transcriptional variation which is associated with immune phenotype and prognosis. Most of the studies published now are grouped by levels of constructed immune scores, seeking differential expression, and then looking for correlation with clinical information. However, to the best of our knowledge, there have been few reports of tumor immune infiltration associated with OS metastatic signatures.

Our primary focus was on the immune infiltration of OS metastases. In our study, interestingly and importantly, we identified that the infiltrations of macrophage and type 2 T-helper cell were decreased in metastatic OS in both two datasets via ssGSEA comparing non-metastasis (Figure 2). Heymann et al. (2019) identified that tumor-associated macrophages (TAMs) and T-lymphocytes are the main TIIC in OS. TAMs promote tumor metastasis through a variety of regulatory pathways (Lin et al., 2019). Interleukin-4 (IL-4) and interleukin-13 (IL-13), Th2-derived cytokines, induce a strong anti-inflammatory phenotype of macrophages, which are also called alternative-activated macrophages (M2) (Guo et al., 2019). It is traditionally believed that M2 macrophages are generally associated with immunosuppression in many tumors (Komohara et al., 2016). Zhou et al. (2017) demonstrated that TAM M2 polarization promoted lung metastasis of OS cells. However, Gomez-Brouchet et al. (2017) reported that contrary to what has been observed in other solid tumors, TAMs (CD163-positive M2-polarized macrophages) are critical in OS progression suppression. Single-cell RNA sequencing (scRNA-seq) has recently developed rapidly, and it can be used to more accurately detect the immune infiltration of OS metastases.

Then, WGCNA based on TARGET-OS and GSE21257 were used to further discover the metastasis-, MA-, and Th2-related module (Figure 3). It was found that genes in lightgreen (TARGET-OS) and brown (GSE21257) had a negative correlation with metastasis, while these genes had a positive correlation with MA and Th2. After modules selected in two datasets, KEGG and GO analyses based on two modules intersected genes indicated that these genes were associated with multiple tumor immune infiltrations (Figure 4). Subsequently, the intersection of hubgenes of metastasis, MA, and Th2 in two datasets was conducted to explore the 11 key metastatic immune-related genes in OS (Figure 4).

Finally, models of OS metastatic immune-related signatures were constructed based on GSE21257 via univariate Cox regression (Table 1), multivariate Cox regression (Figure 5), Lasso Cox regression (Figure 6). In the multivariate Cox model, almost all of the high-risk OS patients developed distant metastases, and only one patient did not develop metastasis. The multivariate Cox hazard ratios of *MS4A7* (1.951, *P*-value: 0.165), *LCP2* (2.231, *P*-value: 0.051), *MNDA* (2.106, *P*-value: 0.064), and *C3AR1* (17.443, *P*-value: 0.011) were all more than 1, which indicated that these genes promote OS metastasis. However, the K–M curves demonstrated that patients with high expression of these four genes had better metastasis-free survival (Supplementary Figure 4). Therefore, considering the sample size is small and the possible influence of outliers on multivariate Cox, we further used the Lasso algorithm. In the lasso results, all patients with high-risk scores in the training cohort, testing cohort, and total cohort had metastases within 5 years. The most crucial thing was that *MSR1* and *TLR7* were common components of univariate Cox, multivariate Cox, and Lasso Cox models. Meanwhile, *MSR1* and *TLR7* were significantly differentially expressed between metastasis and non-metastasis in GSE21257, GSE21257, and TARGET-OS. By conducting metastasis-free and overall survival analyses, these two crucial metastatic immune-related genes, *MSR1* and *TLR7*, were identified not only inhibiting metastasis but also prolonging overall survival in OS (Figure 9). To our knowledge, this is the first time that *MSR1* and *TLR7* have been found in anti-metastasis in OS.

Macrophage Scavenger Receptor 1, MSR1, also known as CD204, is expressed in both M1 and M2 (Martinez et al., 2006). It is a poor prognostic target in non-small cell lung cancer (Li et al., 2018). The MSR1 cluster of KLK14 represents the strongest risk factor identified in non-familial breast cancer and an important risk factor for prostate cancer (Rose et al., 2018). However, Guo et al. (2019) found that triggering MSR1 can induce the activation of JNK in M2 macrophages and the activation of MSR1/JNK signaling pathway leads to M2 polarization to M1 macrophages, which is eliminated in macrophages lacking MSR1. In our work, *MSR1* plays an anti-metastatic role of TIIC in OS. Low-expressed *MSR1* has not only a worse metastasis-free survival probability but also a worse overall survival. Further understanding of the causes of *MSR1* reduction in TIIC of OS and the development of appropriate solutions may benefit the metastasis and survival of OS patients.

The importance of toll-like receptors, TLRs, is now well known as stimulating innate and adaptive immunity. Toll-like receptor 7 (*TLR7*) was mainly expressed in macrophages, plasmacytoid dendritic, NK, and B cells, but not in tumor cells (Trinchieri and Sher, 2007). TLR7 agonists are the only toll-like receptor agonists approved for clinical treatment, although they are currently limited to topical use in various skin cancers (Kobold et al., 2014). Activation of TLR7 and inhibition of TGF-β receptor I (TβRI) reprogrammed tumor-associated macrophages into M1-type macrophages (Peng et al., 2013). In our study, similar to *MSR1*, patients with reduced *TLR7* have lower metastasis-free survival probability and overall survival. How to improve the expression of TLR7 in OS TIIC is the next step that we should further study.

There were some limitations to this study. First, the sample size of each dataset is relatively small, which may cause some bias. Second, only GSE21257 includes the tumor metastasis time of follow-up, we used it to build the model and used the internal data set. Two external data sets, TARGET-OS and GSE42352, could only validate models via metastatic risk scores and genes’ expressions. Although the joint analysis of the three datasets could improve the stability and reliability of the results, a prospective cohort study should be conducted with larger sample size. Thirdly, TNM staging was lost in a large number of samples, so we failed to combine TIIC in OS, *MSR1*, and *TLR7* with tumor stage and grade, which required further verification. Finally, what we have done was only based on transcriptomic data analysis, so further experimental verification and other omics level analyses and cross-validation should be conducted.

Overall, MA and Th2 were significantly reducted in OS metastases, which were analyzed via ssGSEA based on gene markers of 28 TIIC subsets. Afterward, WGCNA based on TARGET-OS and GSE21257 were used to find the most relevant modules with metastasis, MA and Th2. Intersection analysis and univariate/multivariate/Lasso Cox regression were conducted to screen out gene signatures of TIIC associated with metastasis in OS. We found that *MSR1* and *TLR7* had significant effects on anti-metastasis in OS, which may prolong patients’ overall survival. Therefore, to explore in-depth the reasons for the decrease of *MSR1* and *TLR7*, and to develop corresponding countermeasures may make a breakthrough in the prevention and treatment of OS metastasis.

## Data Availability Statement

Publicly available datasets were analyzed in this study. This data can be found here: https://xena.ucsc.edu/ and https://www.ncbi.nlm.nih.gov/geo/.

## Author Contributions

ZC and HH conceived the project and wrote the manuscript. ZC and YW participated in data analysis. FZ participated in the discussion and language editing. ZQ reviewed the manuscript. All authors contributed to the article and approved the submitted version.

## Conflict of Interest

The authors declare that the research was conducted in the absence of any commercial or financial relationships that could be construed as a potential conflict of interest.

## Footnotes

1 https://xenabrowser.net/

## Supplementary Material

Supplementary Figure 1 Detection of outlier and selection of soft power in WGCNA. Soft power selection of TARGET-OS **(A,B)** and GSE21257 **(F,G)**. Top-free detection of TARGET-OS **(C,D)** and GSE21257 **(H,I)**. Detection of an outlier in GSE21257 **(E)**.

Supplementary Figure 2 Correlation analyses between module membership and gene significance in selected modules. Correlations between module membership in the brown module (GSE21257) with gene significance for macrophage **(A)**, type 2 T helper cell **(B)**, and metastasis **(C)**. Correlations between module membership in the lightgreen module (TARGET-OS) with gene significance for macrophage **(D)**, type 2 T helper cell **(E)**, and metastasis **(F)**.

Supplementary Figure 3 11 hubgenes expressions in GSE21257 **(A)**, GSE42352 **(B)**, and TARGET-OS **(C)**. Venn plot: the intersection of 185 modules intersected immune-related genes and 11 selected immune-related hubgenes.

Supplementary Figure 4 The metastasis-free survival K–M curves of seven multivariate-Cox genes in GSE21257.

Supplementary Figure 5 The forest plot of Lasso Cox model.

## Abbreviations

GEO: Gene Expression Omnibus
GO: Gene Ontology
KEGG: Kyoto Encyclopedia of Genes and Genomes
K–M: Kaplan Meier
MA: Macrophage
NES: Normalized enrichment scores
OS: Osteosarcoma
ROC: Receiver operating characteristic
ssGSEA: Single sample Gene Set Enrichment Analysis
TARGET-OS: Therapeutically Applicable Research To Generate Effective Treatments – Osteosarcoma
Th2: Type 2 T helper cell
TIIC: Tumor immune-infiltrating cells
TAMs: Tumor-associated macrophages
TME: Tumor microenvironment
TOM: Topological overlap matrix
WGCNA: Weighted gene co-expression network analysis.
