# Supplementary figures and images for: Identification of Immune-Related Genes MSR1 and TLR7 in Relation to Macrophage and Type-2 T-Helper Cells in Osteosarcoma Tumor Micro-Environments as Anti-metastasis Signatures
Source: Front Mol Biosci. 2020 Dec 14;7:576298. doi: 10.3389/fmolb.2020.576298 (PMC7768026; doi:10.3389/fmolb.2020.576298)

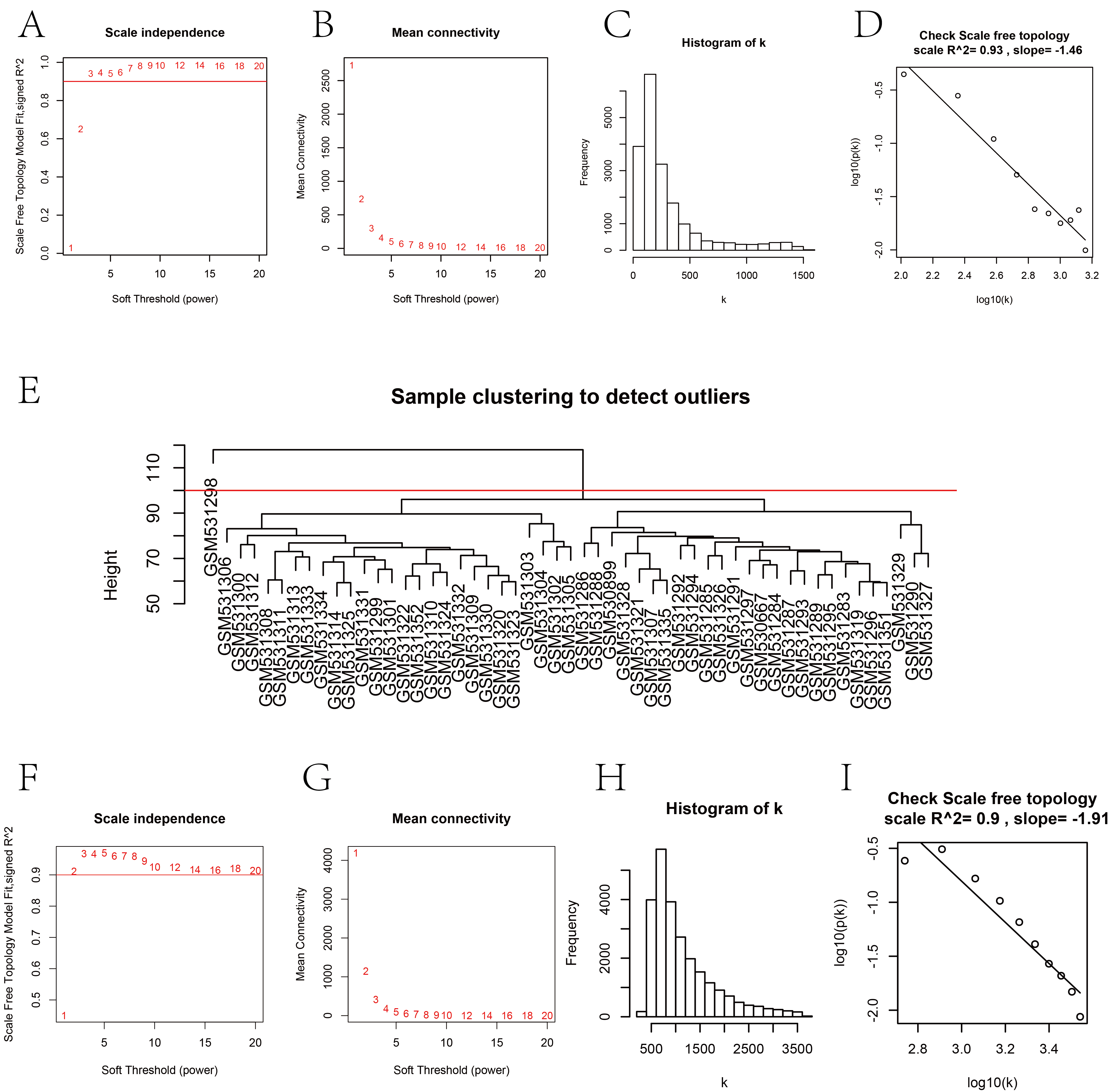

Supplement: Supplementary Figure 1 — Detection of outlier and selection of soft power in WGCNA. Soft power selection of TARGET-OS (A,B) and GSE21257 (F,G). Top-free detection of TARGET-OS (C,D) and GSE21257 (H,I). Detection of an outlier in GSE21257 (E). [file Image_1.tif]

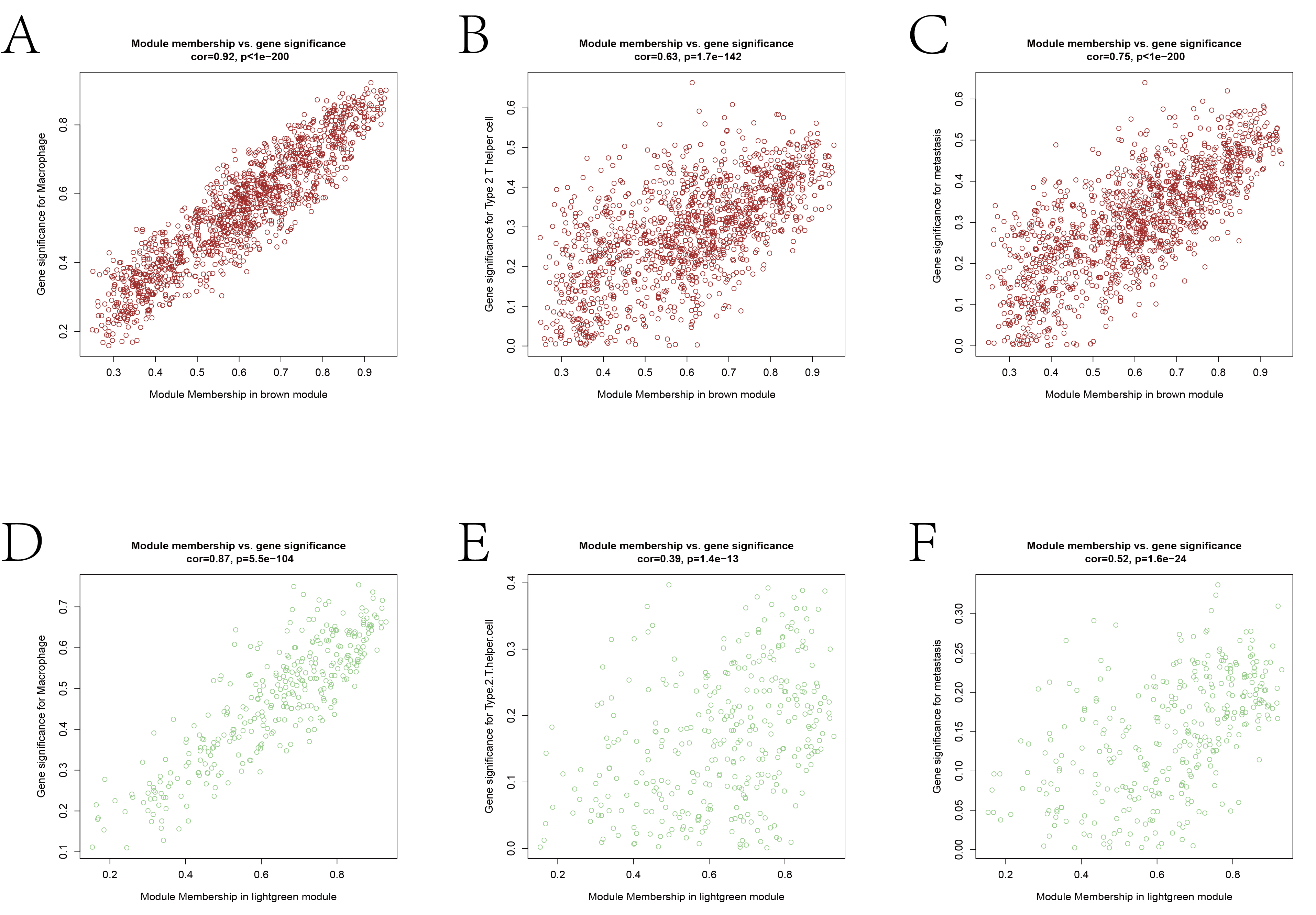

Supplement: Supplementary Figure 2 — Correlation analyses between module membership and gene significance in selected modules. Correlations between module membership in the brown module (GSE21257) with gene significance for macrophage (A), type 2 T helper cell (B), and metastasis (C). Correlations between module membership in the lightgreen module (TARGET-OS) with gene significance for macrophage (D), type 2 T helper cell (E), and metastasis (F). [file Image_2.tif]

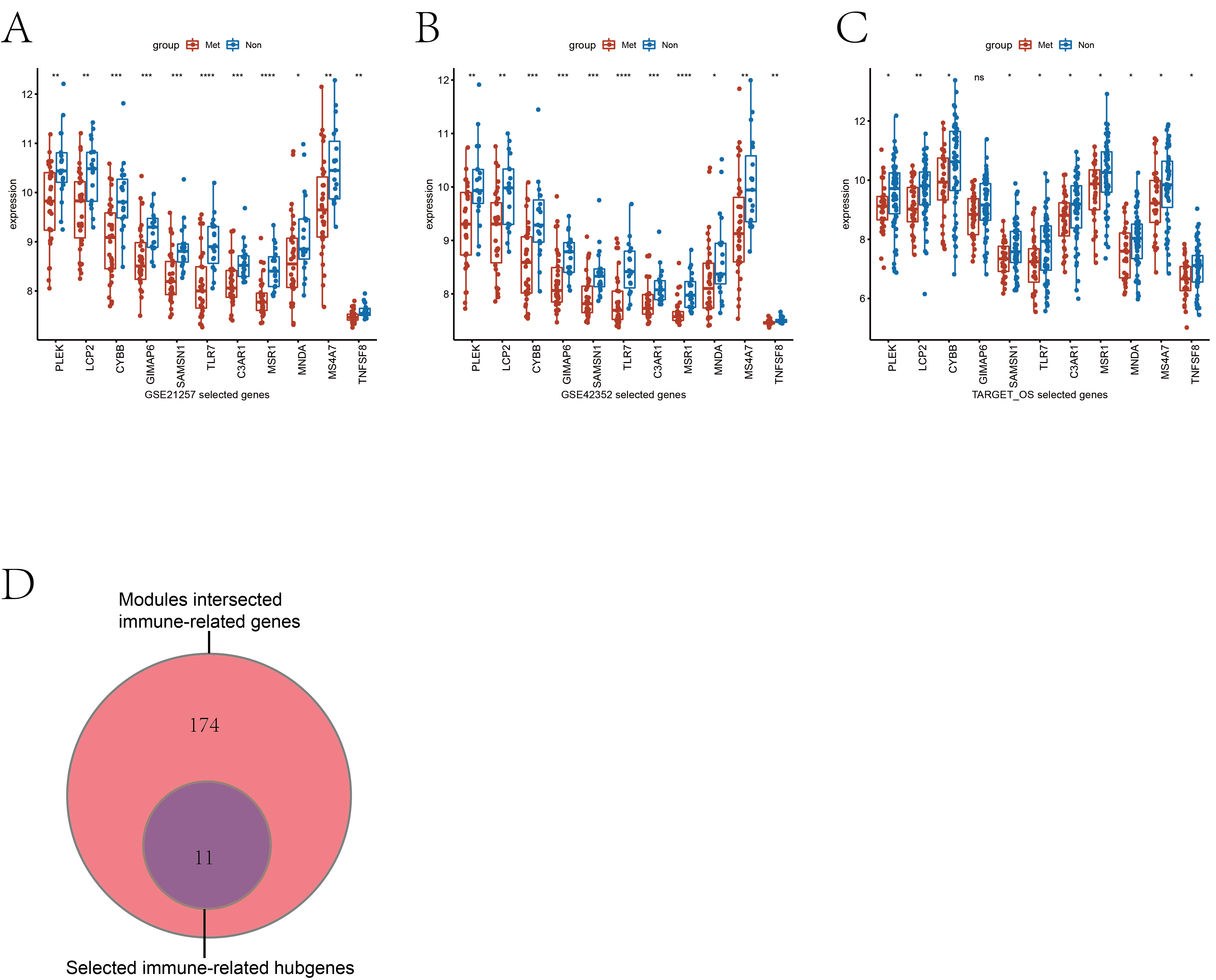

Supplement: Supplementary Figure 3 — 11 hubgenes expressions in GSE21257 (A), GSE42352 (B), and TARGET-OS (C). Venn plot: the intersection of 185 modules intersected immune-related genes and 11 selected immune-related hubgenes. [file Image_3.tif]

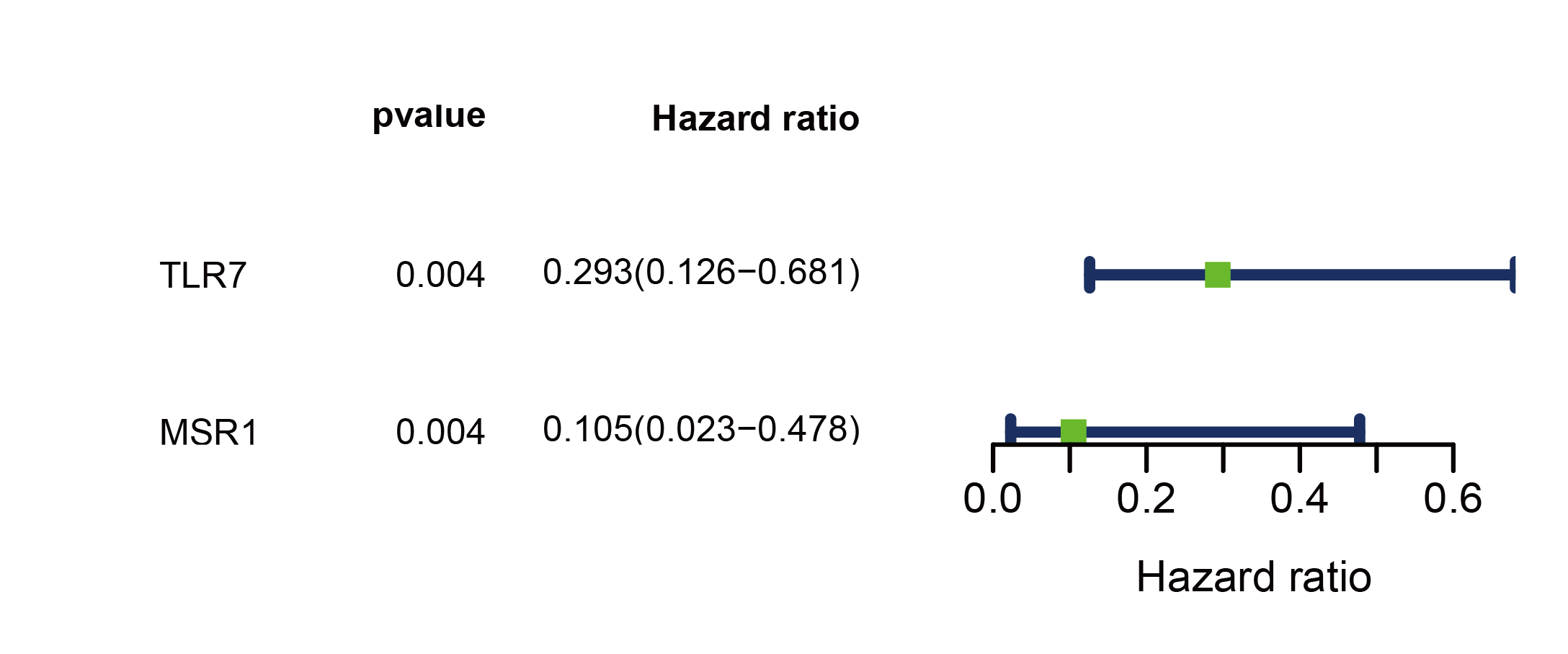

Supplement: Supplementary Figure 5 — The forest plot of Lasso Cox model. [file Image_5.tif]
